# Critical Metal Adsorption by Biotic and Abiotic Hydrous Manganese Oxides: Implications for Acid Mine Drainage Resource Recovery

**DOI:** 10.1021/acsomega.5c04278

**Published:** 2025-09-10

**Authors:** Tashane J. Boothe-Lordon, Rosemary C. Capo, Brian W. Stewart, Travis A. Olds, Carla E. Rosenfeld

**Affiliations:** † Department of Geology and Environmental Science, 6614 University of Pittsburgh, Pittsburgh, Pennsylvania 15260, United States; ‡ 110067 Carnegie Museum of Natural History, Pittsburgh, Pennsylvania 15213, United States

## Abstract

Acid mine drainage (AMD) remediation facilities can produce treatment byproducts with near ore grade concentrations of rare-earth elements (REEs), cobalt, and manganese. High concentrations of these critical metals in treatment solids are often associated with hydrous manganese oxides (HMOs) through adsorption and/or coprecipitation. Chemical and microbial oxidation processes can influence HMO formation, mineralogy, and sorption efficiency. Here, we investigate the adsorption of rare-earth elements and yttrium (REY), cobalt, and nickel over 31 days by (1) abiotic HMO (δ-MnO_2_ and c-disordered H+ birnessite) produced by chemical oxidation and (2) bitotic HMO produced by Mn-oxidizing fungi, *Paraphaeosphaeria sporulosa* and *Stagonospora* sp. After 31 days, ∼70% of REY was adsorbed by abiotic HMO, whereas >99% of REY was adsorbed by biotic HMO and/or fungal biomass within 7 days. Biotic HMO also adsorbed ∼30% Ni and ∼75% Co; however, Co and Ni adsorption by abiotic HMO was negligible. Both biotic and abiotic HMOs were initially poorly crystalline. However, over the course of the experiment, abiotic HMO was transformed to more crystalline phases, resulting in a reduced adsorption capacity and significant desorption of Co and Ni. In contrast, the biotic HMO remained stable and resistant to structural changes over time. This study demonstrates that biotic HMOs are highly efficient at adsorbing Co and REY and that fungal biomass can also play a significant role in this process, particularly for REY.

## Graphical abstract

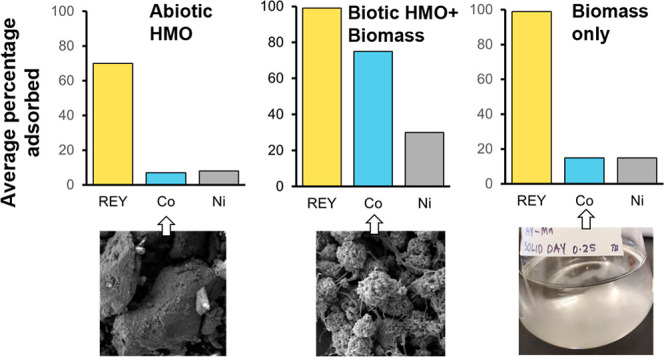

## 1 Introduction

Hydrous manganese oxide (HMO) phases are common in soils, sediments, and aquatic environments, occurring as crusts, coatings, concretions, and nodules.^1–3^ Naturally occurring HMO minerals are typically formed by microbial (e.g., fungal and bacterial) oxidation of Mn­(II) to Mn­(III) and Mn­(IV) via complex catalytic reactions.^4–7^ These minerals are generally disordered, poorly crystalline,^8,9^ and highly reactive due to charge imbalances or vacancies in the crystal structures.^2^ HMOs are environmentally important as they control the aqueous concentration, transport, and bioavailability of multiple metals, contaminants, and organic compounds through oxidation and adsorption.^2,10–14^ Studies show that these minerals, when synthesized in the lab using known Mn-oxidizing bacteria (e.g., *Leptothrix discophora*, *Bacillus* sp., and *Pseudomonas putida*) or fungi (e.g., *Acremonium* sp., *Pyrenochaeta* sp., and *Plectosphaerella cucumerina*), also have a high capacity to adsorb and coprecipitate metals such as Co, Ni, Pb, Se, and Zn.^15–18^ However, chemically synthesized abiotic analogues of natural HMO minerals can exhibit slower reaction kinetics and reduced efficiency in trace metal uptake when compared to biotic HMOs. These differences may stem from variations in chemical and physical properties, such as fewer structural vacancies, altered Mn­(III)/Mn­(IV) molar ratios, and the absence of organic matter (e.g., biomass or extracellular polymeric substances), which results from differing oxidation mechanisms.^19–22^

The “metal scavenging” properties of biotic and abiotic HMOs have been exploited in many environmental applications including agriculture, drinking water purification, toxic metal remediation, and the recovery of radionuclides.^23,24^ The potential use of HMOs in recovering energy-critical metals during acid mine drainage (AMD) treatment has also gained attention due to the high global demand for these metals, the scarcity of ores, and the existing monopoly of supply by few countries.^25–29^

Manganese, Mn­(II), can be a major dissolved species in acid mine drainage (AMD) derived from coal mines. In western Pennsylvania, USA, for example, the average dissolved Mn concentration in AMD (2.35 mg/L) exceeds the Pennsylvania Department of Environmental Protection instream limit of 1.0 mg/L^30^ and can be as high as 74 mg/L.^31^ Precipitated ochres on the beds of AMD-affected streams in this region can have Mn concentrations exceeding 200 mg/kg,^31^ occurring as multiple Mn-oxide and hydroxide mineral phases, including birnessite, pyrolusite, and todorokite.^3,10,32^ The Mn content of AMD treatment precipitates typically averages 600 mg/kg and can exceed 400,000 mg/kg in some passive treatment systems.^28,33^ HMO minerals are thought to play a key role in trace metal attenuation (including energy-critical metals such as REY, Ni, and Co) in AMD treatment systems.^28,32–34^ Concentrations of 500–2000 mg/kg REY and greater than 5000 mg/kg Co in some AMD precipitates in Pennsylvania,^33^ for example, have been attributed to the presence of biotic HMOs, particularly in passive treatment systems.^28,34^

However, not all AMD treatment systems produce precipitates with high critical metal concentrations.^28,33^ In order for AMD treatment solids to become a viable feedstock for critical metals, it is important to not only effectively remove critical metals from solution but also to understand how the biogeochemical conditions in the treatment systems promote their concentration in certain mineral phases. Remaining knowledge gaps include (1) the relative importance of abiotic and biotic HMO in critical metal removal in AMD systems; (2) critical metal attenuation by HMO from multi-element solutions over extended periods (>24 h), reflecting the complex biogeochemical conditions typical of AMD systems; and (3) comparison of REY and trace metal attenuation by HMO. In most cases, studies on REY behavior tend to focus on synthetic HMO minerals or isolate Ce as the REY of interest.

In this study, we examine the attenuation of ten critical metals^35^ (Co, Ni, and the rare-earth elements La, Nd, Ce, Gd, Pr, Dy, Yb, and Y) from solution by HMO produced by two fungal species, *Paraphaeosphaeria sporulosa* (previously *Paraconiothyrium sporulosum*) and *Stagonospora* sp. We also evaluate the importance of the fungal biomass in the sorption of these metals. These fungi are known to be present in AMD treatment systems in western Pennsylvania^36^ and produce forms of HMO such as vernadite (δ-MnO_2_) or birnessite^37^ that are highly disordered and have a high adsorption capacity. We also conducted parallel experiments using lab-synthesized δ-MnO_2_ and c-disordered H+ birnessite (hereafter termed H+ birnessite) to examine the interaction of these abiotic minerals with critical metals. The efficiency of critical metal sorption was assessed by conducting time series analyses of the measured dissolved metal concentrations during the experiment. Critical metal recovery is therefore equivalent to its uptake or removal from solution by the HMO substrate. The mineralogy and structure of abiotic and biotic HMO minerals at different time points during the experiments were characterized using scanning electron microscopy (SEM) and X-ray diffraction (XRD) to identify possible mineral transformation and corresponding changes in metal uptake rates. This study expands on previous studies that investigated AMD treatment precipitates using micro-characterization techniques (e.g., by XPS and micro-XRF).^28,34^ Our findings will also inform future field experiments at AMD treatment sites and will also involve the use of these analytical techniques. Our experiments elucidate the role of biotic and abiotic HMO in adsorbing and concentrating various metals in AMD systems as well as the role of the fungi in this process, with implications for the viability of AMD treatment precipitates as sustainable sources of critical metals.

## 2 Materials and Methods

### 2.1 Biotic HMO Experimental Design

Biotic HMO was produced using two common environmental fungal species, *P. sporulosa* and *Stagonospora* sp., originally isolated from a sewage-contaminated pond and an acid mine drainage (AMD) treatment facility, respectively, and maintained in culture at the Carnegie Museum of Natural History in Pittsburgh, Pennsylvania.^37^ These fungi are known to be less sensitive to heavy metals and high metal concentrations typical of AMD and are able to tolerate >10 mM of Mn­(II).^36^ The fungi were inoculated in triplicate in Erlenmeyer flasks containing 150 mL sterile AY growth media prepared using the method described by Rosenfeld et al.^37^ The growth medium was buffered at pH 7 using HEPES buffer (0.02 M) and amended with approximately 800 μM MnCl_2_ to allow the synthesis of HMO by the fungi. Parallel biosorption experiments were also conducted in which both fungal species were inoculated in growth media without the addition of MnCl_2_ (Table S1). All flasks were stored throughout the experiment at room temperature in the dark to limit photochemical reactions. Following inoculation, the fungi were allowed to grow for 14 days, after which the flasks were spiked with a critical metal solution containing Co, Ni, Y, La, Ce, Pr, Nd, Gd, Dy, and Yb dissolved in 10% nitric acid. The concentration of each metal in the flasks (Table S7) is 70 times higher than the concentration typical of Appalachian AMD.^26^ Immediately following the addition of the critical metal solution, the flasks were swirled to mix the solution. For each biotic experiment, eight additional flasks were also prepared to allow the collection of solid HMO-critical metal precipitates or biomass samples at specific time points throughout the experiment. In addition to the fungal HMO and biosorption experiments, two abiotic control experiments (Table S1) were also prepared in triplicate. One abiotic control experiment contained growth media and dissolved MnCl_2_, while the second abiotic control experiment contained growth media without fungi or MnCl_2_.

#### 2.1.1 Critical Metals and HMO-Biomass Sampling

In most passive AMD treatment systems, Mn minerals are typically armored to limestone or other surface and become immobile unless removed by mechanical methods.^34,38^ Studies by Rosenfeld et al., 2020,^37^ and Xu et al., 2023,^39^ noted partial phase transformation in HMO minerals over 31 days. Based on these findings, we opted to conduct a 31 day experiment to assess how the mineralogy and structure of Mn minerals change with time and how this may affect long-term adsorption and retention of the metals. Critical metal removal by biotic HMO and fungal biomass was determined by measuring the aqueous metal concentrations at each time point. Aliquots (2 mL) were collected from each flask at 8 time points after the addition of the critical metals: 0.05 h, 6 h, 1 d, 4 d, 7 d, 10 d, 18 d, and 31 d. The sampling time points were designed to be more frequent at the onset of the experiments and become less frequent over time. This allows us to capture changes in the critical metal concentrations early in the experiment since adsorption reactions can occur rapidly at pH 7. The lag time between the addition of the critical metals and the retrieval of the first sample (0.05 h) was approximately 2–3 min. The samples were filtered using a 0.22 μm mixed cellulose ester (MCE) filter and stored at −20 °C. HMO precipitates and biomass were harvested from the additional flasks at the same time points by using a vacuum filter with 0.22 μm MCE filters. A sterile plastic spatula was used to dislodge any solids that adhered to the surface of the flask. The filter paper with the solid paste was placed in a small Petri dish, sealed with parafilm, and stored at −20 °C to prevent desiccation and further oxidation reactions.

### 2.2 Abiotic HMO Experimental Design

The structure and crystallinity of synthetic HMO can vary widely due to structural imperfections, method of synthesis, pH, and aging.^3,40,41^ Under microbially mediated conditions in natural and AMD systems, the precipitated hydrous Mn oxide tends to be finer grained, and the structure tends to be less crystalline and more disordered.^15,42,43^ These structural characteristics, as well as environmental conditions, can result in anomalies in the XRD patterns such as missing peaks, split peaks, and broad peaks. The HMO minerals H+ birnessite and δ-MnO_2_ are known to be produced naturally by *P. sporulosa* and *Stagonospora* sp.^37^ To best approximate the actual HMO substrate produced by these fungi in an AMD treatment system, we used the method of Hinkle et al.^41^ to synthesize H+ birnessite and δ-MnO_2_.

The experimental conditions of the biotic experiments described above were replicated in the abiotic experiments by suspending approximately 9.2 mg of H+ birnessite and δ-MnO_2_ in 150 mL of sterile AY growth media, buffered at pH 7. The solutions were equilibrated for 1 h before adding critical metal solution. Each experiment assessing dissolved metal concentrations was conducted in triplicate and proceeded for 31 days at room temperature in a dark environment with nine additional flasks, from which solid-phase abiotic HMO-critical metal precipitates were harvested. Sampling of the critical metal-growth medium solution and abiotic HMO solids was conducted in a similar manner to the biotic experiments at nine time points after the addition of the critical metals: 0.05 h, 3 h, 6 h, 1 d, 4 d, 7 d, 10 d, 18 d, and 31 d. Samples were also stored in a manner similar to the biotic experiments.

### 2.3 Analytical Techniques

Filtered solution samples (2 mL) collected from all flasks were acid-digested by using 300 μL of concentrated trace-grade nitric acid in trace metal-free centrifuge tubes. Samples were allowed to react with the concentrated HNO_3_ for 2 h at 60 °C before diluting with distilled–deionized water (dd-H_2_O) to 10 mL (final HNO_3_ concentration of 3%). Samples were analyzed using a Thermo iCAP Q inductively coupled plasma mass spectrometer (ICP-MS) at Northwestern University Quantitative Bulk-Elemental Information Core (QBIC). Blank samples containing 3% nitric acid and dd-H_2_O only were analyzed for quality control. The internal standards were matrix matched and consisted of 1 ng/mL of In and Bi. Instrument performance was optimized prior to the start of each run and monitored to ensure that there were no memory effects throughout the run. Further details on the methodology and data processing are provided in the Supporting Information.

Powder X-ray diffraction (PXRD) and scanning electron microscopy (SEM) analyses were conducted at the Carnegie Museum of Natural History, Pittsburgh. PXRD data for air-dried biotic and abiotic HMO samples were obtained using a Bruker Apex II Single-Crystal X-ray Diffractometer (SCXRD) equipped with an air-cooled IμS 2.0 microfocus source (Mo Kα radiation, λ = 0.71075 Å, 50 kV, 40 mA) and a Photon III CPAD detector. Identifications were made using the PDF 5+ International Centre for Diffraction Data (ICDD) database, paired with Materials Data (MDI) Jade Pro software. A pseudo-Gandolfi-like motion was used to randomize diffraction from each sample, which had an average volume of ∼2 × 10^–3^ mm^3^. The observed *d*-values and intensities were derived by full-profile fitting using a JADE Pro. SEM imaging was done on a Tescan Vega II XMU variable pressure instrument with an Oxford Instruments INCA Energy 250XT Energy-Dispersive X-ray Analysis system. Air-dried biotic samples were gold coated using a Denton Vacuum Desk V Sample Preparation system.

## 3 Results and Discussion

### 3.1 Abiotic and Biotic HMO Products

The abiotically synthesized HMOs, δ-MnO_2_ and H^+^ birnessite, have layered, angular structures (Figure S1). PXRD patterns of δ-MnO_2_ and H^+^ birnessite prior to the experiments show broad symmetrical peaks at 29.3° 2θ and 17° 2θ corresponding to the (310)/(020) and (200)/(110) planes, respectively. There is also a broadening and apparent splitting of the peaks at ∼7.6° 2θ (Figure 1A). For H+ birnessite, the characteristic 002 reflection expected at 11.3° 2θ ^2,44^ is absent. Additionally, for δ-MnO_2_, the basal peak at 5.5° 2θ forms a shoulder, but this is absent in the H^+^ birnessite pattern. These shifts and irregularities in the basal reflections (00*l*) of the abiotic HMOs relative to other reported XRD (e.g., Drits et al.^45^) have also been reported in previous studies,^42^ underscoring the high levels of disorder in the sheet stacking arrangement and variations in interlayer compositions typical of phyllomanganates.

**Figure 1:**
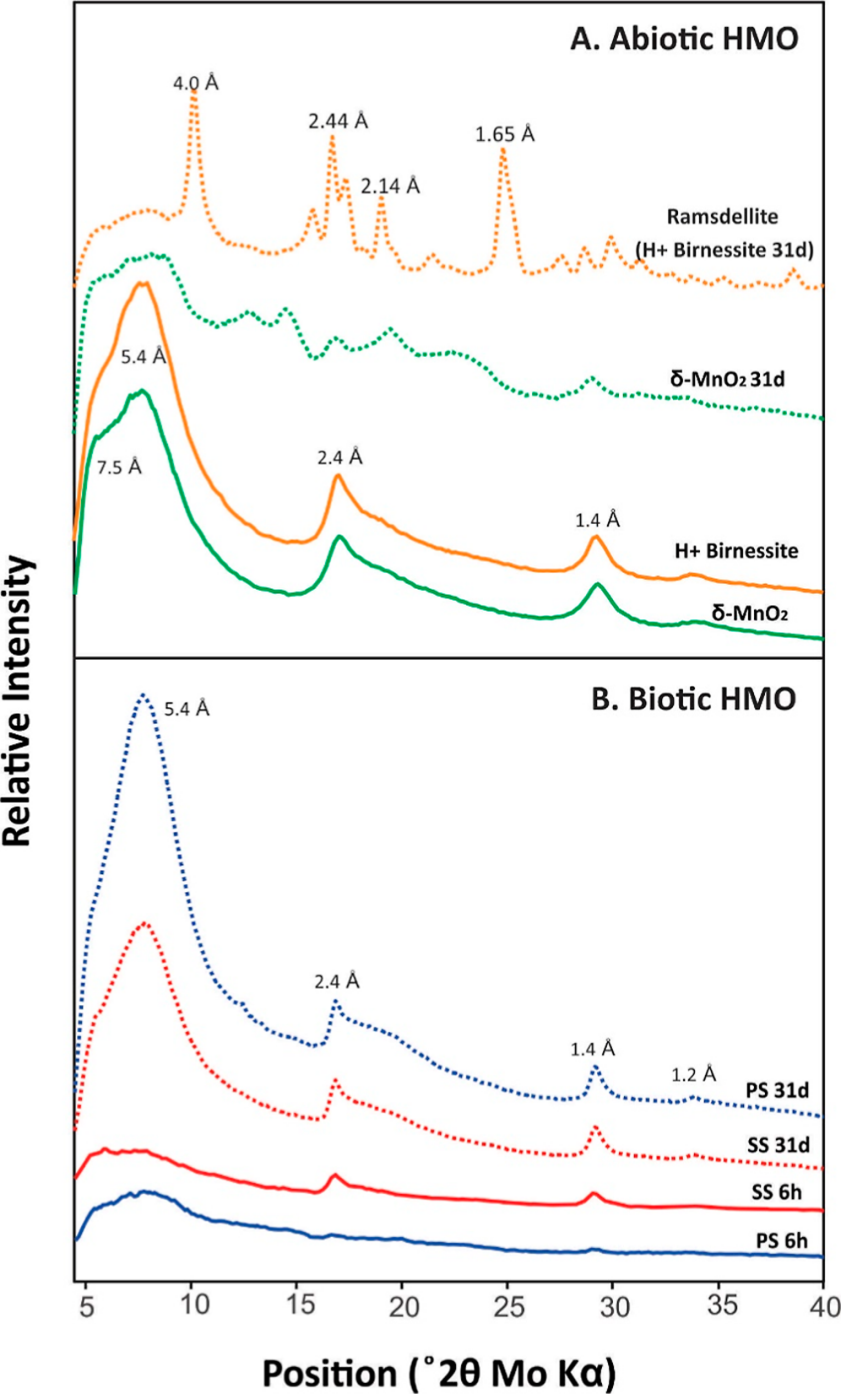
(A) X-ray diffraction (XRD) pattern of abiotic HMO δ-MnO_2_ and H+ birnessite before critical metal addition and 31 days after critical metal addition (δ-MnO_2_ 31d and H+ birnessite 31d) metals. (B) XRD patterns of biotic HMO produced by *P. sporulosa* retrieved at 6 h (PS 6h) and 31 days (PS 31d) and *Stagonospora* sp. retrieved at 6 h (SS 6h) and 31 days (SS 31d).

After 31 days, both abiotic HMOs underwent phase transformation. δ-MnO_2_ appears to have been transformed into a multiphase solid that is not readily identifiable. H^+^ birnessite was transformed to ramsdellite as shown by the major peak positions at 24.8° 2θ, 19° 2θ, 16.7° 2θ, and 10.15° 2θ (Figure 1a). Ramsdellite is a tunnel structure Mn-oxide formed by linking adjacent single chains to form double chains^46^ and is composed of Mn­(IV).^47^ Aging, as well as interactions with other cations, can transform layered HMO to more crystalline tunnel varieties.^13,37,39^ Given the increase in Mn­(II) in solution (Figure S6), we hypothesize that a partial reduction of Mn­(IV) in H+ birnessite by Ce, Co, and/or interaction with the HEPES buffer resulted in the formation of Mn­(III) which subsequently underwent disproportionation to produce Mn­(IV) and aqueous Mn­(II).

The biotic HMOs produced by *Stagonospora* sp. and *P. sporulosa* in these experiments are poorly crystalline; however, unlike the abiotic HMO, they remained relatively stable over the duration of the experiment. Both fungi produced an amorphous solid or a poorly crystalline phyllomanganate resembling vernadite (or its synthetic analogue, δ-MnO_2_) with very weak peaks at 29.3° and 17° 2θ (Figure 1B). Aging and cation interaction did not result in significant mineral transformations. After 31 days, the biotic HMO products showed stronger peaks at 29.3 2θ and 17° 2θ corresponding to the in layer (310)/(020) and (200)/(110) planes, respectively. δ-MnO_2_ is produced by both bacteria and fungi in the presence of Mn­(II).^13,37,39,48^ Crystals of this mineral phase occur as exceptionally thin sheets, typically less than 100 nm in length. These sheets exhibit strong disorder, manifested as random stacking sequences of Mn–O sheets and variably populated interlayer cation and H_2_O content, resulting in weak, broad, or absent (001) and (002) reflections.^2^ For all samples studied here, the (001) basal peak at 5.4° 2θ is largely absent, confirming the presence of small crystals and high levels of disorder. The broad peak at 7.8° 2θ is possibly due to the presence of chitin in the fungal cell wall.^37,39^ These XRD patterns suggest that fungal δ-MnO_2_ remained stable and highly reactive throughout the experiment and may explain the high sorption capacity of the biotic HMO.

Although the surface area of HMO minerals can be variable depending on the synthesis method,^23^ biotic HMOs tend to have a larger surface area than abiotic HMO.^15,49^ However, surface area is not expected to be a limiting factor for critical metal adsorption in these experiments, given the concentration of Mn used in our experiments (∼800 μM) and reported surface area and site density of biotic and abiotic HMO.^50^ Nevertheless, the behavior of the biotic and abiotic HMO minerals during these experiments could be constrained in future work by using controls, such as abiotic oxides aged in media without critical metal additions, abiotic HMO amended with Mn­(II), or abiotic HMO incubated with biomass. Similarly, the use of alternative growth media with a lower organic carbon content could help eliminate potential interactions between organic molecules and dissolved metals.

The HMO precipitates that formed through fungal oxidation were associated with the fungal biomass, either directly on the hyphae or in the interstitial (extracellular) areas (Figure 2). HMO precipitated by *P. sporulosa* encrusts the entire length of the hyphae (Figure 2a,b) and is similar to the morphology of HMO produced by the fungus *P. cucumerina*.^51^ Unlike *P. sporulosa*, HMO produced by *Stagonospora* sp. occurs as larger, spherical structures (approximately 30 μm) adjacent to the fungal hyphae. The HMO produced by both fungi consists of an aggregation of randomly oriented plate-like structures with erose edges, similar to those described in previous studies.^49,51–53^ This gives the biotic HMO a sponge-like or crumpled appearance very dissimilar to the blocky, angular grains of the abiotic HMOs (Figure S1).

**Figure 2:**
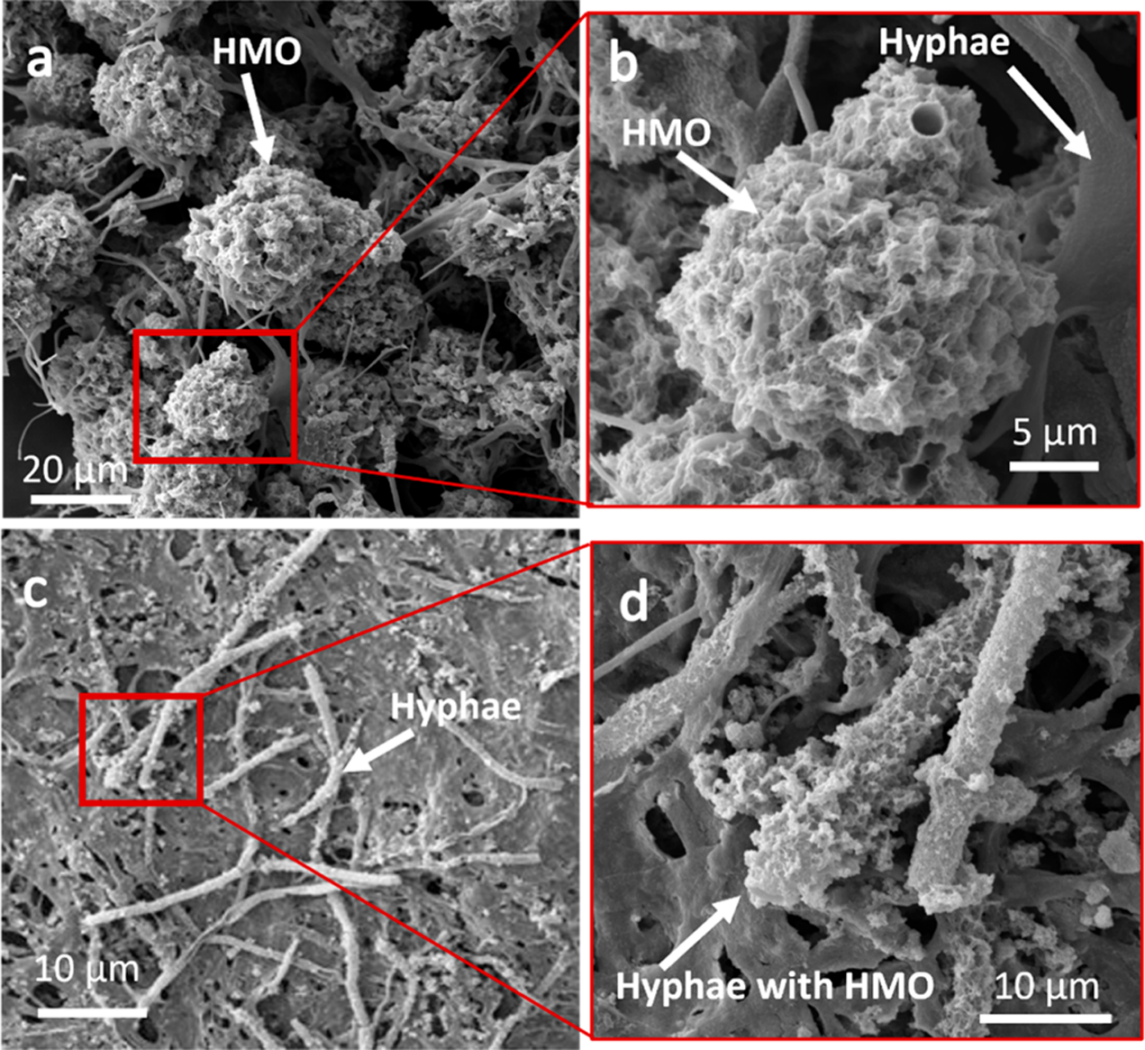
(a,b) Scanning electron microscopy (SEM) images of *Stagonospora* sp. biomass retrieved at 31 days showing the occurrence and associations of HMO minerals. (c,d) SEM images of *P. sporulosa* retrieved at 31 days showing the occurrence and associations of HMO minerals.

Previous studies have shown that *Stagonospora* sp. and *P. sporulosa* produce HMO with Mn­(III) and Mn­(IV) content varying from 80% to 97%.^37,51,54,55^ Fungi oxidize Mn­(II) via hyphae-associated superoxide production,^56^ by the production of cell–wall-associated enzymes such as multicopper oxidase^52^ and by cell-free secretomes (biomolecules) mediated by extracellular proteins.^57^ While it is unclear whether these processes occur concurrently, the precipitation of hyphal-associated HMO (as in the case of *P. sporulosa*) suggests that superoxide production and cell wall enzymes may be the dominant mechanisms for Mn­(II) oxidation. Oxidation of Mn­(II) by extracellular proteins could explain the location of HMO in the interstitial area of the biomass as seen with *Stagonospora* sp. This difference in the morphology and location of the biotic HMO has been cited as evidence that the mechanism for fungal Mn-oxidation can vary among species.^58^

*Stagonospora* sp. and *P. sporulosa* showed a marked difference in the amount of dissolved Mn oxidized to form HMO. Over the 14 day growth period, *Stagonospora* sp. oxidized approximately 30% more dissolved Mn than *P. sporulosa* (Figure S2). This variation in Mn-oxidation capacity may be attributed to differences in growth rates, metabolic processes, or oxidation mechanisms involving reactive oxygen species, proteins, and enzymes.^52,56,57^ The effects of the growth rate on Mn-oxidation could be assessed by normalizing HMO production to biomass produced. However, because the biotic HMO is strongly bound by, and enmeshed in, the fungal biomass, we were not able to directly measure the fungal biomass.

Throughout the experiments, dissolved Mn content fluctuated as a response to interactions of HMO with metals (Figure S2). Following the addition of critical metals, dissolved Mn concentrations increased from almost undetectable (2.5 μM) to 60 μM in the *Stagonospora* sp. experiment over 31 days. Conversely, in the *P. sporulosa* experiment, dissolved Mn concentrations declined after the addition of critical metals, from 270 to 180 μM representing an additional 11% decrease in the concentration of dissolved Mn over 31 days. These changes in aqueous Mn­(II) concentrations reflect the complex redox reactions occurring between previously formed HMO and the critical metals, particularly Co­(II) and Ce­(III). Co­(II) and Ce­(III) are readily oxidized by Mn­(IV),^15,16,25,39,59^ producing Mn­(II) through the disproportionation of Mn­(III) ions.^43,44^ The continuous, gradual decline in Mn­(II) concentrations in the *P. sporulosa* experiment suggests that the presence of the critical metals may have slowed but not completely inhibited Mn­(II) adsorption and/or oxidation. Notably, previously precipitated Mn surfaces can catalyze Mn­(II) adsorption through heterogeneous oxidation.^38,60^ The adsorbed ions may eventually be oxidized, thus increasing the amount of Mn minerals over time. However, the presence of the Co, Ni, and the REE in solution with Mn­(II) introduces potential competition for adsorption sites.^17,18^

### 3.2 Adsorption of Transition Metals Co and Ni by HMO

The abiotic HMOs used in our experiments were relatively inefficient at sorbing Co and Ni. Initially, there was a steady decline in Co and Ni concentrations in both the δ-MnO_2_ and H+ birnessite experiments, with maximum adsorption of these metals occurring on day 4 (29% and 19% Co adsorbed, respectively, and 24% and 18% Ni adsorbed, respectively (Figure 3a,b)). Subsequently, Co and Ni concentrations in both experiments increased due to desorption. Overall, the adsorption of Co and Ni in these experiments was minimal over 31 days, with δ-MnO_2_ adsorbing 4.5% of the metals, while H+ birnessite removed approximately 11% of the metals.

**Figure 3:**
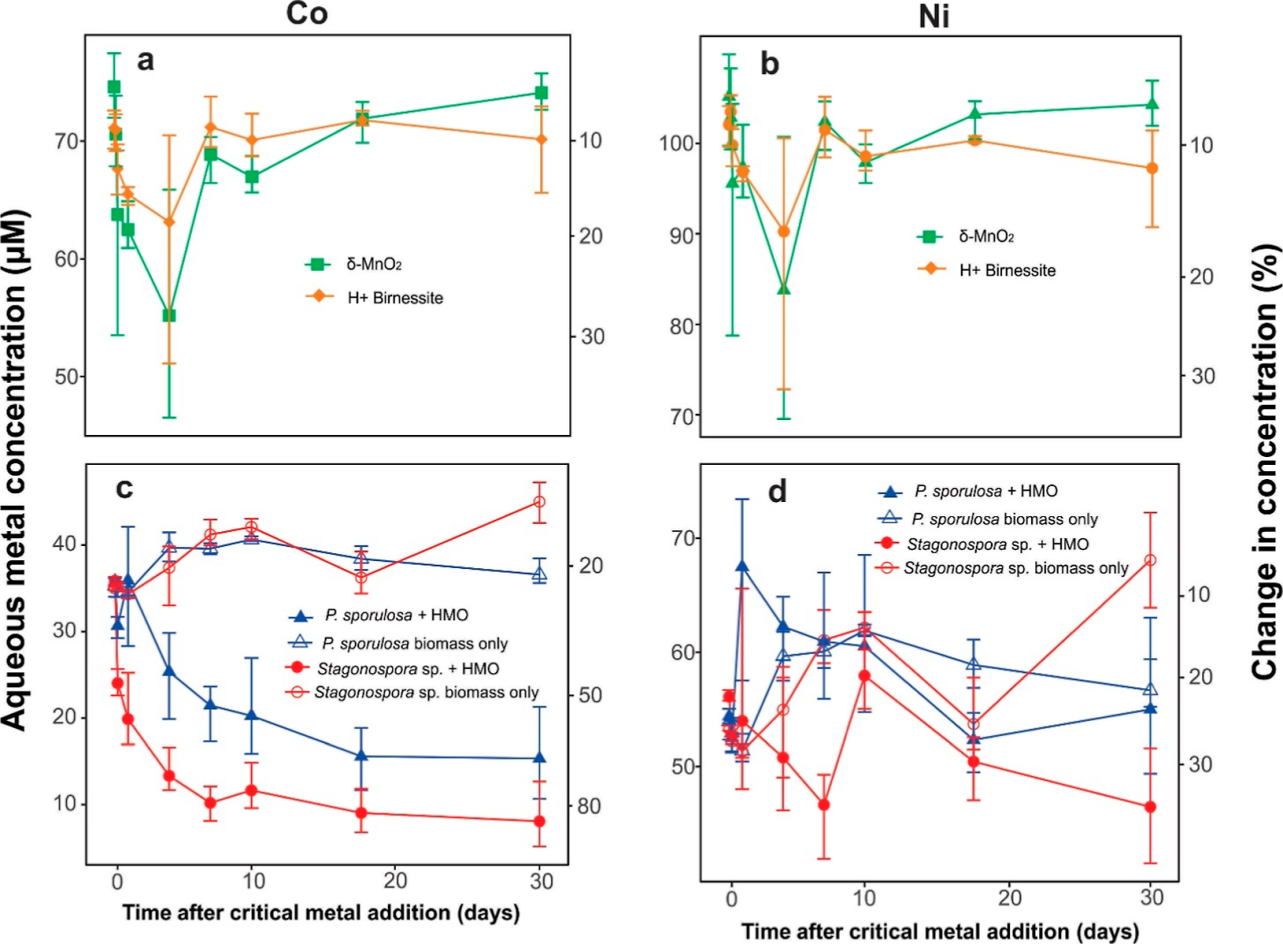
Change in dissolved Co and Ni concentrations in the presence of: (a,b) abiotic HMO, δ-MnO_2_, and H+ birnessite; (c,d) biotic HMO produced by *Stagonospora* sp. and *P. sporulosa* and/or *Stagonospora* sp. and *P. sporulosa* biomass. Each point represents the average value for three replicate experiments. The top and bottom of the error bar represent the maximum and minimum values, respectively.

The low adsorption of Co and Ni by the abiotic HMO may be due to the transformation of δ-MnO_2_ and H+ birnessite into secondary or tunnel crystalline HMO phases. Tunnel structure HMO minerals, such as ramsdellite, are highly crystalline and have been reported to exhibit very low Co and Ni absorption in multimetallic solutions.^16,43^ In contrast, synthetic H+ birnessite and δ-MnO_2_, which possess a layered or sheet-like structure, readily adsorb Co and Ni in monometallic solutions.^15,17,61^ In multimetal solutions, however, these synthetic layer-type HMOs adsorb significantly less metals.^17,61,62^ This highlights not only the importance of the types of HMO phases but also the influence of competing metal ions on adsorption efficiency.

Biotic HMOs produced by *Stagonospora* sp. and *P. sporulosa* are very efficient at adsorbing Co (Figure 3c). Dissolved Co concentrations declined rapidly and steadily in the *Stagonospora* sp. experiment, resulting in greater than 80% Co adsorbed over 31 days. In the *P. sporulosa*-HMO experiment, Co concentrations decreased less rapidly in the first 6 h. Desorption and subsequent fluctuation in Co concentrations in the first day of the experiment resulted in 12% less Co adsorbed than HMO-*Stagonospora* sp.

HMO is known to readily adsorb Co due to strong redox reactions with Co­(II).^17,43,63^ Up to 80–90% of Co adsorbed on HMO can be present as Co­(III) or Co­(IV).^10,15,39^ The poorly crystalline, vacancy-rich biotic HMO products of *Stagonospora* sp. and *P. sporulosa* are likely responsible for the rapid adsorption of Co observed in these experiments, as Co­(III) is typically incorporated into layer vacancies in the HMO structure^17,43,53^ or at corner- and edge-sharing sites.^39,43^ Importantly, studies also show that Co will fill edge sites preferentially before diffusing to layer vacancies.^63^ This has implications for other trace metals such as Ni that are also adsorbed at the edge sites or incorporated into the HMO structure.

Over 31 days, HMO produced by *Stagonospora* sp. adsorbed 12% more Ni than did HMO-*P. sporulosa* (Figure 3d). However, for both biotic HMO experiments, the amount of Ni adsorbed after 31 days was not significantly different from values recorded within the first day of the experiment. This is because Ni concentrations fluctuated widely, particularly between day 1 and day 10, reflecting considerable amounts of desorption. Less Ni was adsorbed by biotic HMO (3.15 μmole) compared to Co (5.55 μmole), even though the concentration of Ni was 1.5 times greater than Co in the spiking solution. This is consistent with previous studies that also show preferential adsorption of Co over Ni.^16,43,50^ While Ni may be incorporated into layer vacancies,^18,64^ most studies report that Ni is adsorbed near layer vacancies to form corner-sharing Ni­(II) species in the HMO structure or at edge sites.^18,43,61,65^ However, as discussed previously, Co will fill edge sites preferentially before diffusing to layer vacancies^63^ and Ni does not compete with Co for these edge sites.^43^ Thus, the presence of Co can result in three times lower adsorption of Ni onto HMO.^16^

Because the biotic HMO is associated with the fungal biomass, there is potential that biomass contributes to the adsorption of Co and Ni. However, this study did not identify significant biosorption of Co or Ni in the biomass-only experiments (Figure 3c,d). *P. sporulosa* biomass removed approximately 20% of Co and Ni, whereas *Stagonospora* sp. biomass removed less than 10% of these metals. This indicates that biomass alone does not contribute significantly to adsorption in these experiments and underscores the importance of the biotic HMO in controlling metal adsorption, particularly Co. The lower uptake of Co and Ni by the biomass, compared to that of the biotic HMO, may be attributed to differences in the surface area and the quantity and types of sorption sites. Previous studies show mixed results on Co and Ni biosorption, with some reporting minimal adsorption by fungal biomass^39,43,49^ and others reporting significant Co and Ni biosorption by fungal^66–68^ and bacterial biomass.^69,70^ This suggests that the sorption capacity can vary significantly among microbial species, experimental conditions, and possibly the availability of binding sites in multimetallic or monometallic solutions.

### 3.3 Adsorption of REY by HMO

The REYs exhibited higher adsorption rates than Co and Ni in all experiments The abiotic HMOs adsorbed approximately 70–73% of the REYs by day 4 of the experiment. Like Co and Ni, desorption of adsorbed REY was observed on day 4 (Figure 4a), likely due to the phase changes in the abiotic HMO. However, in this case, REY desorption was minimal, with 68–71% of the REY remaining adsorbed after 31 days.

**Figure 4:**
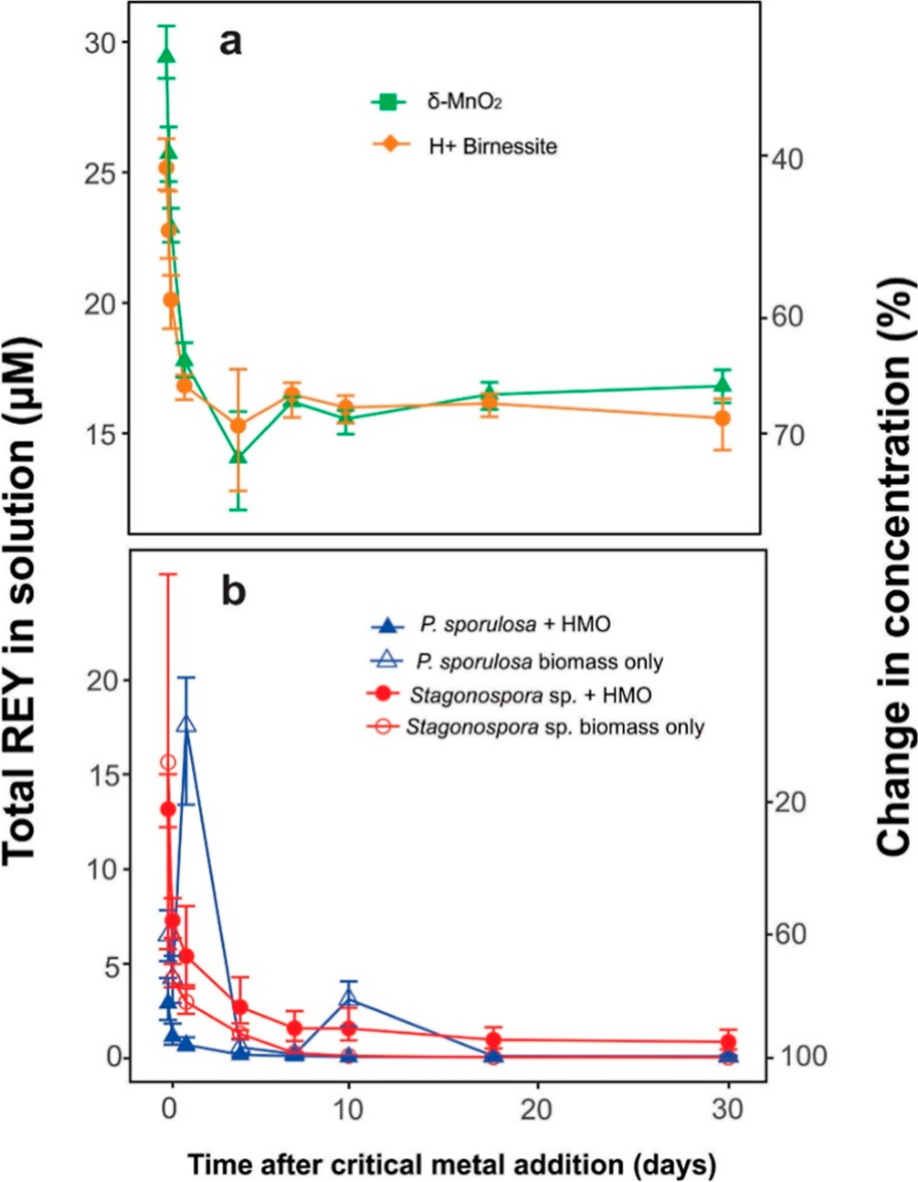
Change in dissolved Co, Ni, and total rare-earth element concentrations in the presence of (a) abiotic HMO and (b) biotic HMO and/or fungal biomass after the addition of critical metals. Each point represents the average value for three replicate experiments. The top and bottom of the error bar represent the maximum and minimum values, respectively.

In contrast, all REYs were rapidly adsorbed in the biotic systems (Figure 4b). HMO-*P. sporulosa* was more efficient than HMO-*Stagonospora* sp., with greater than 90% of total REY adsorbed within 6 h. HMO-*Stagonospora* sp. adsorbed 90% of the total REY within 7 days. Overall, the REYs are more readily adsorbed than Co and Ni, with over 99–99.9% adsorption over 31 days. Interestingly, HMO-*Stagonospora* sp., which was the most efficient at adsorbing Co and Ni, adsorbed less REY than did HMO-*P. sporulosa*. However, given the high REY adsorption observed in the biomass-only experiments (discussed below), it is likely that REY adsorption by the biotic HMO in these experiments includes a contribution from the biomass.

The high adsorption efficiency of REYs observed in these experiments (>99%) is typical of AMD treatment systems which generally have efficiencies exceeding 90%.^34^ In normal surface water, REE can form complexes with a variety of anions (e.g., SO_4_^2–^, CO_3_^2–^, OH^–^)^71–73^ as well as organic compounds.^74,75^ In AMD systems, however, SO_4_^2–^ is dominant and can form complexes with 50 to 70% of REY. If SO_4_^2–^ concentrations are lower, free REE ions dominate (>90%).^72^ REE as free ions or aqueous complexes is adsorbed by Mn and other mineral phases during treatment. Based on the composition of biotic and abiotic HMO experiments, REE is potentially complexed with OH^–^ ions and/or organic molecules or present as free ions.

Substantial amounts of REYs were removed through biosorption, in contrast to our observations for Ni and Co. REY concentrations decreased rapidly in the presence of *P. sporulosa* and *Stagonospora* sp. biomass at rate similar to, or greater than those observed in the biotic HMO experiments (Figure 4b), demonstrating that fungal biomass can serve as an effective sorbent for REYs.

The process of biosorption does not typically result in the oxidation of adsorbed metals. For example, redox-sensitive metals such as Ce and Co associated with bacterial and fungal cells remain in their +3 and +2 states, respectively, suggesting that biosorption inhibits oxidation.^15,25,66,76^ Instead of oxidation, biosorption occurs through ion exchange, complexation, or electrostatic interactions between metal ions and functional groups on the cell wall.^77,78^ These functional groups originate from chitin and other polysaccharides and glycoproteins such as glucans and mannans in the fungal cell wall.^77,79^ The specific functional groups involved in biosorption for an individual species are difficult to ascertain, especially for fungi;^79^ however, certain groups are known to play a key role. For instance, carboxyl and phosphate functional groups are known to facilitate Eu­(II) adsorption by bacteria,^80^ and sulfhydryl sites have been shown to be the dominant adsorption sites for some divalent ions at environmentally significant concentrations.^81^

The biotic HMO, biomass-only, and abiotic HMO systems display preferential adsorption of light rare-earth elements (LREEs) compared with the middle rare-earth elements (MREEs) or heavy rare-earth elements (HREEs) (Figure 5). This difference is more pronounced in the HMO-*Stagonospora* sp. and *Stagonospora* sp. biomass experiments after 31 days (Figure 6), with roughly 1 order of magnitude more LREE adsorbed than HREE. Preferential adsorption of LREE by HMO has previously been observed experimentally,^59^ in natural AMD precipitates^34^ and in groundwater systems.^82^ The fungal species involved in the experiment also appear to influence the REE patterns. The experiments involving *Stagonospora* sp. display a very similar concave LREE pattern, whereas the *P. sporulosa* experiments display a relatively flat LREE pattern.

**Figure 5:**
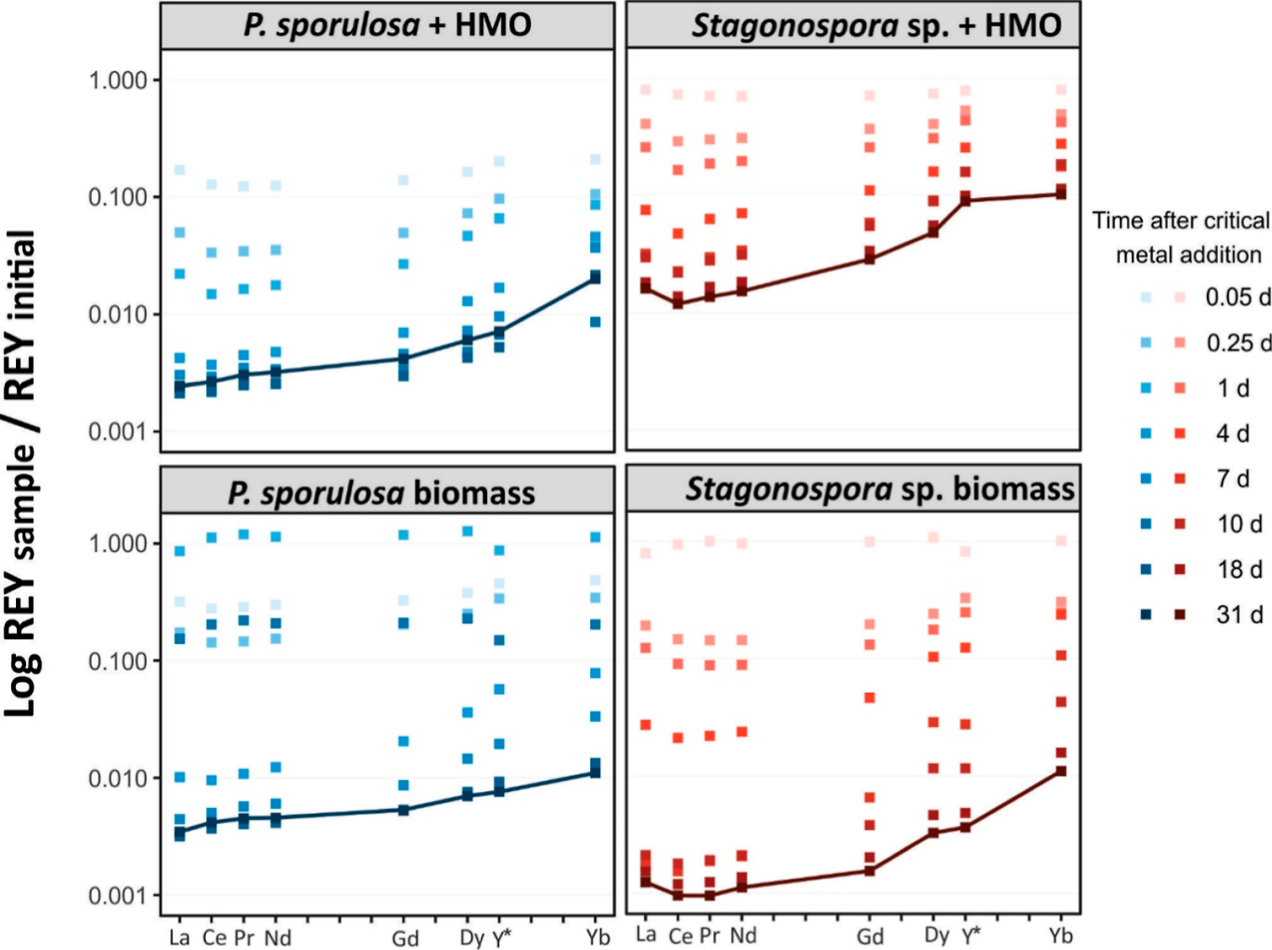
Ratio of REY in solution at each sample point to the initial concentration in the flask. Yttrium is plotted in the position of Ho (not analyzed) due to its nearly identical ionic (3+) radius. Biotic HMO with biomass, and fungal biomass only removed greater than 99% after 6 h and greater than 99.9% after 7 days. Each point represents the average value for three replicate experiments.

**Figure 6:**
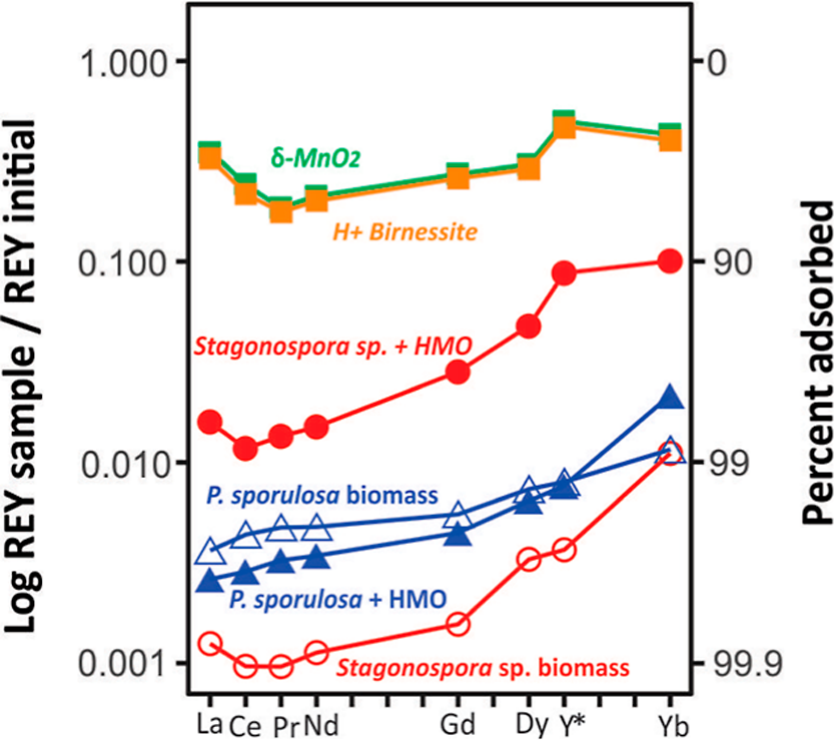
Comparison of the REY patterns after 31 days. Ratio of REY in solution at each sample point to the initial concentration in the flask.

Despite the preferential adsorption of LREE, we note an apparent lack of Ce anomalies in all experiments (Figure 6). Cerium is readily oxidized by HMO, which could lead to preferential Ce incorporation in the HMO mineral. This process reduces the concentration of Ce in the solution relative to its neighboring REYs, creating a negative Ce anomaly (low normalized Ce concentration relative to other REYs).^25,59^ Weak or absent Ce anomalies for all experimental conditions may imply that significant Ce­(III) oxidation did not occur. However, it is possible that the anomaly was erased by the rapid uptake of all the LREEs at pH 7.^83^ Cerium anomalies may be more likely in acidic solutions because at these conditions, other trivalent REY ions are inhibited from being adsorbed onto HMO.^73,76^ Experiments have also shown that REY can form complexes with organic compounds before being adsorbed to HMO, which can also prevent Ce anomalies from developing.^74,83^

## 4 Environmental Implications

HMO in AMD treatment solids is disproportionately enriched with REY relative to other common metal oxides, such as hydrous Fe and Al oxides.^33,34^ Manganese in AMD can be difficult to treat and is best removed at pH >9 using strong oxidants.^30,84^ However, in passive AMD treatment systems that rely on natural chemical and microbial processes, microbes, including the fungi explored in this study, mediate the precipitation of HMO at around pH 6–7.^36,85^ These treatment systems, which are typically characterized by gradual pH increases, allow for selective precipitation of Fe, Al, and Mn solids based on their pH-related solubilities. Since the adsorption edge of REEs is between pH 4.5 and 7, they are primarily adsorbed at pH 3.5–5 by Al minerals as these minerals precipitate and by Mn minerals during Mn precipitation at pH 6–7.^34,86,87^ This highlights the importance of HMO phases in treatment solids for REY adsorption at a circumneutral pH.

Our experiments demonstrate that at neutral pH, the biotic HMO rapidly adsorbs approximately 75% of Co and 99.9% of REY over 31 days, with most of the uptake occurring within the first 7 days. In contrast, only 30% of Ni was adsorbed by biotic HMO. Meanwhile, abiotic HMO adsorbed 70% of REY over the course of the experiment, but Co and Ni adsorption were negligible. Both biotic and abiotic HMOs are poorly crystalline. However, the biotic HMOs, which were identified as δ-MnO_2_, exhibited greater stability and resistance to structural changes over time when compared to abiotic δ-MnO_2_ and H+ birnessite formed through chemical oxidation. The stability of the biotic HMO may be related to ongoing Mn redox cycling facilitated by the fungi, which helps to maintain the highly reactive nature of the biotic HMO over time. In contrast, the abiotic HMOs undergo structural changes to produce ramsdellite and a mixed-phase Mn mineral. These mineral phase changes and increases in crystallinity are accompanied by desorption at around day 4 of the experiments, resulting in considerably less adsorption capacity.

Microbes in AMD treatment systems (including fungi and bacteria) may be associated with mineral surfaces or other suspended organic solids.^88^ Microbial biomass associated with minerals, as well as those that do not directly oxidize Mn, may provide additional surfaces on which adsorption can occur since studies show that biomass does not compete with or prevent adsorption of trace metals by HMO.^89,90^ Our study shows that fungi have varying biosorption capacities or affinities for different metals. This alludes to potential competition or preference by these metals for functional groups on the cell walls of the fungi. Fungal biomass, in this study, rapidly adsorbed 90–99.9% of REY, indicating a strong affinity for these elements. Because fungal biomass appears to preferentially accumulate REY over Co and Ni, there is the potential for exploiting biosorption as a mechanism for selective REY recovery. However, the long-term fate of critical metals adsorbed on biomass remains unclear. Some studies have shown desorption of REY^25,91^ and the dissociation of REY-organic ligand bonds over time,^74^ potentially allowing these metals to become adsorbed and/or oxidized by hydrous metal oxides. REY adsorbed to biomass on surfaces may eventually become incorporated within the HMO structure and other oxides after death of the fungi and decomposition of the biomass. In contrast, critical metals adsorbed to microbes that are suspended in the water column may not become concentrated in the treatment precipitates, posing challenges to recovery.

Our results show that a biotic HMO is more effective than abiotic HMO for adsorbing all critical metals assessed in the study. This suggests that biotic HMOs in passive treatment systems are promising targets for critical metal recovery. Optimizing microbial HMO precipitation in these systems can allow efficient remediation of Mn while promoting the adsorption and concentration of critical metals, such as Co and REYs. These findings are particularly relevant to AMD discharges with high Mn content that are adjusted to circumneutral pH by using passive treatment technology. At this pH, Mn is the dominant major metal, and the precipitation of HMO minerals is expected to be mediated by microbes, especially fungi.^36^ Elevated Mn concentrations have been documented in coal- ^26,31,33^ and ore-related AMD discharges worldwide.^92,93^ Under these conditions, similar trends in the critical metal behavior are expected.

The demand for technology and energy-critical metals is estimated to increase exponentially within the next 10 to 15 years.^35,94,95^ There is therefore an immediate need to stabilize the global supply chain of these metals to reduce the risks of economic disruptions.^35^ The elevated concentrations of REY, Co, and Mn in some AMD treatment precipitates may make them an attractive unconventional source of these critical metals that can potentially be exploited while alleviating the environmental degradation associated with traditional mining.^33^ The potential economic and environmental benefits associated with using AMD treatment precipitates as critical metal feedstocks could incentivize the development of new and more efficient remediation systems.

## Supplementary Material





## Notes

Details of analytical procedure; detection limits; data treatment; concentration of critical metals in spiking solution; SEM of δ-MnO_2_ and H+ birnessite; change in aqueous Mn concentration in biotic experiments; change in aqueous Co concentration in the control experiments; change in aqueous Ni concentration in the control experiments; change in aqueous total REY concentration in the control experiments; and Mn concentrations in abiotic HMO experiments (PDF)
Experimental conditions; results of biotic HMO experiment; results of abiotic HMO experiment; results of biomass-only experiment; and results of control experiment (XLSX)

## References

[1] Miyata N., Tani Y., Sakata M., Iwahori K. (2007). Microbial manganese
oxide formation and interaction with toxic metal ions. J. Biosci. Bioeng..

[2] Villalobos M., Toner B., Bargar J., Sposito G. (2003). Characterization of
the manganese oxide produced by Pseudomonas putida strain MnB1. Geochim. Cosmochim. Acta.

[3] Bargar J. R., Fuller C. C., Marcus M. A., Brearley A. J., Perez
De la Rosa M., Webb S. M., Caldwell W. A. (2009). Structural characterization
of terrestrial microbial Mn oxides from Pinal Creek, AZ. Geochim. Cosmochim. Acta.

[4] Nealson, K. H. ; Tebo, B. M. ; Rosson, R. A. Occurrence and Mechanisms of Microbial Oxidation of Manganese. Advances in Applied Microbiology; Elsevier, 1988; Vol. 33; pp 279–318.

[5] Brouwers G. J., Vijgenboom E., Corstjens P., De Vrind J., De Vrind-De
Jong E. (2000). Bacterial Mn2+ oxidizing systems and multicopper oxidases: an overview
of mechanisms and functions. Geomicrobiol. J..

[6] Tebo B. M. (1991). Manganese
(II) oxidation in the suboxic zone of the Black Sea. Deep-Sea Res., Part A.

[7] Ehrlich H. L. (1999). Microbes
as geologic agents: their role in mineral formation. Geomicrobiol. J..

[8] Spiro T. G., Bargar J. R., Sposito G., Tebo B. M. (2010). Bacteriogenic manganese
oxides. Acc. Chem. Res..

[9] Li Y., Xu Z., Ma H., S Hursthouse A. (2019). Removal of Manganese (II) from acid
mine wastewater: A review of the challenges and opportunities with
special emphasis on mn-oxidizing bacteria and microalgae. Water.

[10] Kay J. T., Conklin M. H., Fuller C. C., O’Day P. A. (2001). Processes
of nickel and cobalt uptake by a manganese oxide forming sediment
in Pinal Creek, Globe Mining District, Arizona. Environ. Sci. Technol..

[11] Fuller C. C., Harvey J. W. (2000). Reactive uptake of trace metals in the hyporheic zone
of a mining-contaminated stream, Pinal Creek, Arizona. Environ. Sci. Technol..

[12] Moffett J. W., Ho J. (1996). Oxidation of cobalt and manganese in seawater via a common microbially
catalyzed pathway. Geochim. Cosmochim. Acta.

[13] Bargar J. R., Tebo B. M., Bergmann U., Webb S. M., Glatzel P., Chiu V. Q., Villalobos M. (2005). Biotic and
abiotic products of Mn
(II) oxidation by spores of the marine Bacillus sp. strain SG-1. Am. Mineral..

[14] Hausladen D. M., Fendorf S. (2017). Hexavalent chromium generation within naturally structured
soils and sediments. Environ. Sci. Technol..

[15] Tanaka K., Yu Q., Sasaki K., Ohnuki T. (2013). Cobalt (II) oxidation by biogenic
Mn oxide produced by Pseudomonas sp. strain NGY-1. Geomicrobiol. J..

[16] Tani Y., Ohashi M., Miyata N., Seyama H., Iwahori K., Soma M. (2004). Sorption of Co (II), Ni (II), and
Zn (II) on biogenic manganese oxides
produced by a Mn-oxidizing fungus, strain KR21–2. J. Environ. Sci. Health, Part A: Toxic/Hazard. Subst. Environ.
Eng..

[17] Zhao H., Feng X., Lee S., Reinhart B., Elzinga E. J. (2023). Sorption
and oxidation of Co (II) at the surface of birnessite: Impacts of
aqueous Mn (II). Chem. Geol..

[18] Hinkle M. A., Dye K. G., Catalano J. G. (2017). Impact of Mn (II)-manganese oxide
reactions on Ni and Zn speciation. Environ.
Sci. Technol..

[19] Zhu M., Farrow C. L., Post J. E., Livi K. J., Billinge S. J., Ginder-Vogel M., Sparks D. L. (2012). Structural study of biotic and abiotic
poorly-crystalline manganese oxides using atomic pair distribution
function analysis. Geochim. Cosmochim. Acta.

[20] Webb S. M., Tebo B., Bargar J. (2005). Structural
characterization of biogenic
Mn oxides produced in seawater by the marine Bacillus sp. strain SG-1. Am. Mineral..

[21] Nelson Y. M., Lion L. W., Ghiorse W. C., Shuler M. L. (1999). Production of biogenic
Mn oxides by Leptothrix discophora SS-1 in a chemically defined growth
medium and evaluation of their Pb adsorption characteristics. Appl. Environ. Microbiol..

[22] Tebo B. M., Bargar J. R., Clement B. G., Dick G. J., Murray K. J., Parker D., Verity R., Webb S. M. (2004). Biogenic
manganese
oxides: properties and mechanisms of formation. Annu. Rev. Earth Planet. Sci..

[23] Zhou H., Fu C. (2020). Manganese-oxidizing
microbes and biogenic manganese oxides: characterization,
Mn (II) oxidation mechanism and environmental relevance. Rev. Environ. Sci. Bio/Technol..

[24] Szlamkowicz I., Stanberry J., Lugo K., Murphy Z., Ruiz Garcia M., Hunley L., Qafoku N. P., Anagnostopoulos V. (2022). Role of Manganese
Oxides in Controlling Subsurface Metals and Radionuclides Mobility:
A Review. ACS Earth Space Chem..

[25] Ohnuki T., Ozaki T., Kozai N., Nankawa T., Sakamoto F., Sakai T., Suzuki Y., Francis A. (2008). Concurrent transformation
of Ce (III) and formation of biogenic manganese oxides. Chem. Geol..

[26] Stewart B. W., Capo R. C., Hedin B. C., Hedin R. S. (2017). Rare earth
element
resources in coal mine drainage and treatment precipitates in the
Appalachian Basin, USA. Int. J. Coal Geol..

[27] Prudêncio M., Valente T., Marques R., Braga M. S., Pamplona J. (2017). Rare earth
elements, iron and manganese in ochre-precipitates and wetland soils
of a passive treatment system for acid mine drainage. Procedia Earth Planet. Sci..

[28] Hedin B. C., Capo R. C., Stewart B. W., Hedin R. S., Lopano C. L., Stuckman M. Y. (2019). The evaluation of critical rare earth
element (REE)
enriched treatment solids from coal mine drainage passive treatment
systems. Int. J. Coal Geol..

[29] Wallrich I. L., Stewart B. W., Capo R. C., Hedin B. C., Phan T. T. (2020). Neodymium
isotopes track sources of rare earth elements in acidic mine waters. Geochim. Cosmochim. Acta.

[30] Hedin R. S., Wolfe N., Hedin B. (2024). Passive Removal of
Mn from Mine Water
Using Oxic Aggregate Beds: Results of Two Pilot Systems. Mine Water Environ..

[31] Cravotta C. A. (2008). Dissolved metals and associated constituents
in abandoned
coal-mine discharges, Pennsylvania, USA. Part 1: Constituent quantities
and correlations. Appl. Geochem..

[32] Tan H., Zhang G., Heaney P. J., Webb S. M., Burgos W. D. (2010). Characterization
of manganese oxide precipitates from Appalachian coal mine drainage
treatment systems. Appl. Geochem..

[33] Hedin B. C., Hedin R. S., Capo R. C., Stewart B. W. (2020). Critical metal recovery
potential of Appalachian acid mine drainage treatment solids. Int. J. Coal Geol..

[34] Hedin B. C., Stuckman M. Y., Cravotta C. A., Lopano C. L., Capo R. C. (2024). Determination and
prediction of micro scale rare earth
element geochemical associations in mine drainage treatment wastes. Chemosphere.

[35] DOE . Critical Metal Assessement; United States Department of Energy, 2023.

[36] Santelli C. M., Pfister D. H., Lazarus D., Sun L., Burgos W. D., Hansel C. M. (2010). Promotion of Mn (II) oxidation and
remediation of coal
mine drainage in passive treatment systems by diverse fungal and bacterial
communities. Appl. Environ. Microbiol..

[37] Rosenfeld C. E., Sabuda M. C., Hinkle M. A., James B. R., Santelli C. M. (2020). A fungal-mediated
cryptic selenium cycle linked to manganese biogeochemistry. Environ. Sci. Technol..

[38] Luan F., Santelli C. M., Hansel C. M., Burgos W. D. (2012). Defining manganese
(II) removal processes in passive coal mine drainage treatment systems
through laboratory incubation experiments. Appl.
Geochem..

[39] Xu T., Roepke E. W., Flynn E. D., Rosenfeld C. E., Balgooyen S., Ginder-Vogel M., Schuler C. J., Santelli C. M. (2023). Aqueous
Co removal by mycogenic Mn oxides from simulated mining wastewaters. Chemosphere.

[40] Drits V. A., Lanson B., Gaillot A.-C. (2007). Birnessite
polytype systematics and
identification by powder X-ray diffraction. Am. Mineral..

[41] Hinkle M. A., Flynn E. D., Catalano J. G. (2016). Structural
response of phyllomanganates
to wet aging and aqueous Mn (II). Geochim. Cosmochim.
Acta.

[42] Villalobos M., Lanson B., Manceau A., Toner B., Sposito G. (2006). Structural
model for the biogenic Mn oxide produced by Pseudomonas putida. Am. Mineral..

[43] Sasaki K., Kaseyama T., Hirajima T. (2009). Selective sorption of Co2+ over Ni2+
using biogenic manganese oxides. Mater. Trans..

[44] Crowther D. L., Dillard J. G., Murray J. W. (1983). The mechanisms
of Co (II) oxidation
on synthetic birnessite. Geochim. Cosmochim.
Acta.

[45] Drits V. A., Silvester E., Gorshkov A. I., Manceau A. (1997). Structure of synthetic
monoclinic Na-rich birnessite and hexagonal birnessite: I. Results
from X-ray diffraction and selected-area electron diffraction. Am. Mineral..

[46] Post J. E. (1999). Manganese
oxide minerals: Crystal structures and economic and environmental
significance. Proc. Natl. Acad. Sci. U.S.A..

[47] Post J. E., Heaney P. J. (2004). Neutron and synchrotron
X-ray diffraction study of
the structures and dehydration behaviors of ramsdellite and “groutellite”. Am. Mineral..

[48] Miyata N., Maruo K., Tani Y., Tsuno H., Seyama H., Soma M., Iwahori K. (2006). Production of biogenic manganese
oxides by anamorphic ascomycete fungi isolated from streambed pebbles. Geomicrobiol. J..

[49] Sasaki K., Matsuda M., Urata T., Hirajima T., Konno H. (2008). Sorption of
Co2+ ions on the biogenic Mn oxide produced by a Mn-oxidizing fungus,
Paraconiothyrium sp. WL-2. Mater. Trans..

[50] Li Y., Zhao X., Wu J., Gu X. (2020). Surface complexation
modeling of divalent metal cation adsorption on birnessite. Chem. Geol..

[51] Santelli C. M., Webb S. M., Dohnalkova A. C., Hansel C. M. (2011). Diversity of Mn
oxides produced by Mn (II)-oxidizing fungi. Geochim. Cosmochim. Acta.

[52] Miyata N., Tani Y., Maruo K., Tsuno H., Sakata M., Iwahori K. (2006). Manganese (IV) oxide production by
Acremonium sp. strain
KR21–2 and extracellular Mn (II) oxidase activity. Appl. Environ. Microbiol..

[53] Yin H., Li H., Wang Y., Ginder-Vogel M., Qiu G., Feng X., Zheng L., Liu F. (2014). Effects of Co and Ni co-doping on
the structure and reactivity of hexagonal birnessite. Chem. Geol..

[54] Liu X., Dong H., Hansel C. M. (2021). Coupled
Mn (II) and Cr (III) oxidation
mediated by Ascomycete fungi. Environ. Sci.
Technol..

[55] Hinkle M. A., Post J. E., Peralta J., Santelli C. M. (2023). Impacts of sulfonic
acids on fungal manganese oxide production. Geochim. Cosmochim. Acta.

[56] Hansel C. M., Zeiner C. A., Santelli C. M., Webb S. M. (2012). Mn (II) oxidation
by an ascomycete fungus is linked to superoxide production during
asexual reproduction. Proc. Natl. Acad. Sci.
U.S.A..

[57] Zeiner C. A., Purvine S. O., Zink E., Wu S., Paša-Tolić L., Chaput D. L., Santelli C. M., Hansel C. M. (2021). Mechanisms of manganese
(II) oxidation by filamentous ascomycete fungi vary with species and
time as a function of secretome composition. Front. Microbiol..

[58] Tang Y., Zeiner C. A., Santelli C. M., Hansel C. M. (2013). Fungal oxidative
dissolution of the Mn (II)-bearing mineral rhodochrosite and the role
of metabolites in manganese oxide formation. Environ. Microbiol..

[59] Ohta A., Kawabe I. (2001). REE (III) adsorption onto Mn dioxide (δ-MnO2)
and Fe oxyhydroxide: Ce (III) oxidation by δ-MnO2. Geochim. Cosmochim. Acta.

[60] Cravotta C. A. (2021). Interactive PHREEQ-N-AMDTreat water-quality
modeling
tools to evaluate performance and design of treatment systems for
acid mine drainage. Appl. Geochem..

[61] Lefkowitz J. P., Elzinga E. J. (2017). Structural alteration
of hexagonal birnessite by aqueous
Mn (II): Impacts on Ni (II) sorption. Chem.
Geol..

[62] Lefkowitz J. P., Elzinga E. J. (2015). Impacts of aqueous
Mn (II) on the sorption of Zn (II)
by hexagonal birnessite. Environ. Sci. Technol..

[63] Wang Y., Benkaddour S., Marafatto F. F., Peña J. (2018). Diffusion-and
pH-dependent reactivity of layer-type MnO2: reactions at particle
edges versus vacancy sites. Environ. Sci. Technol..

[64] Peña J., Kwon K. D., Refson K., Bargar J. R., Sposito G. (2010). Mechanisms
of nickel sorption by a bacteriogenic birnessite. Geochim. Cosmochim. Acta.

[65] Simanova A. A., Kwon K. D., Bone S. E., Bargar J. R., Refson K., Sposito G., Peña J. (2015). Probing the
sorption reactivity of
the edge surfaces in birnessite nanoparticles using nickel (II). Geochim. Cosmochim. Acta.

[66] Sundararaju S., Manjula A., Kumaravel V., Muneeswaran T., Vennila T. (2020). Biosorption of nickel ions using
fungal biomass Penicillium
sp. MRF1 for the treatment of nickel electroplating industrial effluent. Biomass Convers. Biorefin..

[67] Cárdenas
González J. F., Rodríguez Pérez A. S., Vargas Morales J. M., Martínez Juárez V. M., Rodríguez I. A., Cuello C. M., Fonseca G. G., Escalera
Chávez M. E., Muñoz Morales A. (2019). Bioremoval of cobalt
(II) from aqueous solution by three different and resistant fungal
biomasses. Bioinorg. Chem. Appl..

[68] Dusengemungu L., Kasali G., Gwanama C., Ouma K. O. (2020). Recent advances
in biosorption of copper and cobalt by filamentous fungi. Front. Microbiol..

[69] Díaz A., Marrero J., Cabrera G., Coto O., Gómez J. M. (2022). Biosorption
of nickel, cobalt, zinc and copper ions by Serratia marcescens strain
16 in mono and multimetallic systems. Biodegradation.

[70] Valentine N., Bolton Jr H., Kingsley M., Drake G., Balkwill D., Plymale A. (1996). Biosorption
of cadmium, cobalt, nickel, and strontium
by a Bacillus simplex strain isolated from the vadose zone. J. Ind. Microbiol. Biotechnol..

[71] Luo Y.-R., Byrne R. H. (2004). Carbonate complexation of yttrium and the rare earth
elements in natural waters. Geochim. Cosmochim.
Acta.

[72] Serrano M. J. G., Sanz L. F. A., Nordstrom D. K. (2000). REE speciation
in low-temperature
acidic waters and the competitive effects of aluminum. Chem. Geol..

[73] Pourret O., Davranche M. (2013). Rare earth element sorption onto
hydrous manganese
oxide: A modeling study. J. Colloid Interface
Sci..

[74] Davranche M., Pourret O., Gruau G., Dia A., Jin D., Gaertner D. (2008). Competitive binding of REE to humic
acid and manganese
oxide: Impact of reaction kinetics on development of cerium anomaly
and REE adsorption. Chem. Geol..

[75] Marsac R., Davranche M., Gruau G., Bouhnik-Le Coz M., Dia A. (2011). An improved description
of the interactions between rare earth elements
and humic acids by modeling: PHREEQC-Model VI coupling. Geochim. Cosmochim. Acta.

[76] Tanaka K., Tani Y., Takahashi Y., Tanimizu M., Suzuki Y., Kozai N., Ohnuki T. (2010). A specific
Ce oxidation process during
sorption of rare earth elements on biogenic Mn oxide produced by Acremonium
sp. strain KR21–2. Geochim. Cosmochim.
Acta.

[77] Dhankhar R., Hooda A. (2011). Fungal biosorption–an alternative to meet the challenges of
heavy metal pollution in aqueous solutions. Environ. Technol..

[78] Giese E. C. (2020). Biosorption
as green technology for the recovery and separation of rare earth
elements. World J. Microbiol. Biotechnol..

[79] Sun, J. ; Ji, Y. ; Cai, F. ; Li, J. Heavy metal removal through biosorptive pathways. Advances in Water Treatment and Pollution Prevention; Springer, 2012; pp 95–145.

[80] Markai S., Andres Y., Montavon G., Grambow B. (2003). Study of the interaction
between europium (III) and Bacillus subtilis: fixation sites, biosorption
modeling and reversibility. J. Colloid Interface
Sci..

[81] Fein J. B., Yu Q., Nam J., Yee N. (2019). Bacterial cell envelope and extracellular
sulfhydryl binding sites: their roles in metal binding and bioavailability. Chem. Geol..

[82] Liu H., Pourret O., Guo H., Martinez R. E., Zouhri L. (2018). Impact of
hydrous manganese and ferric oxides on the behavior of aqueous rare
earth elements (REE): Evidence from a modeling approach and implication
for the sink of REE. Int. J. Environ. Res. Public
Health.

[83] Davranche M., Pourret O., Gruau G., Dia A., Le Coz-Bouhnik M. (2005). Adsorption
of REE (III)-humate complexes onto MnO2: Experimental evidence for
cerium anomaly and lanthanide tetrad effect suppression. Geochim. Cosmochim. Acta.

[84] Skousen J. G., Ziemkiewicz P. F., McDonald L. M. (2019). Acid mine drainage
formation, control
and treatment: Approaches and strategies. Extr.
Ind. Soc..

[85] Skousen J., Zipper C. E., Rose A., Ziemkiewicz P. F., Nairn R., McDonald L. M., Kleinmann R. L. (2017). Review
of passive systems for acid mine drainage treatment. Mine Water Environ..

[86] Ayora C., Macías F., Torres E., Lozano A., Carrero S., Nieto J.-M., Pérez-López R., Fernández-Martínez A., Castillo-Michel H. (2016). Recovery of rare earth elements and yttrium from passive-remediation
systems of acid mine drainage. Environ. Sci.
Technol..

[87] Lozano A., Ayora C., Fernández-Martínez A. (2019). Sorption of
rare earth elements onto basaluminite: the role of sulfate and pH. Geochim. Cosmochim. Acta.

[88] Robbins E., Cravotta C., Savela C., Nord G. (1999). Hydrobiogeochemical interactions
in anoxic’
limestone drains for neutralization of acidic mine drainage. Fuel.

[89] Peña J., Bargar J. R., Sposito G. (2011). Role of bacterial
biomass in the
sorption of Ni by biomass-birnessite assemblages. Environ. Sci. Technol..

[90] Toner B., Manceau A., Webb S. M., Sposito G. (2006). Zinc sorption to biogenic
hexagonal-birnessite particles within a hydrated bacterial biofilm. Geochim. Cosmochim. Acta.

[91] Takahashi Y., Châtellier X., Hattori K. H., Kato K., Fortin D. (2005). Adsorption
of rare earth elements onto bacterial cell walls and its implication
for REE sorption onto natural microbial mats. Chem. Geol..

[92] Neculita C. M., Rosa E. (2019). A review of the implications
and challenges of manganese removal
from mine drainage. Chemosphere.

[93] Royer-Lavallée A., Neculita C., Coudert L. (2020). Removal and potential recovery of
rare earth elements from mine water. J. Ind.
Eng. Chem..

[94] Alonso E., Sherman A. M., Wallington T. J., Everson M. P., Field F. R., Roth R., Kirchain R. E. (2012). Evaluating
rare earth element availability:
A case with revolutionary demand from clean technologies. Environ. Sci. Technol..

[95] Watari T., Nansai K., Nakajima K. (2020). Review of critical metal dynamics
to 2050 for 48 elements. Resour., Conserv. Recycl..

