# Supplementary material for: Critical Metal Adsorption by Biotic and Abiotic Hydrous Manganese Oxides: Implications for Acid Mine Drainage Resource Recovery
Source: ACS Omega. 2025 Sep 10;10(37):42577–88. doi: 10.1021/acsomega.5c04278 (PMC12461413; doi:10.1021/acsomega.5c04278)
Supplement: Supplementary file 1 [file ao5c04278_si_001.pdf]

Supplementary material for:

CRITICAL METAL ADSORPTION BY BIOTIC AND ABIOTIC HYDROUS MANGANESE OXIDES: IMPLICATIONS FOR ACID MINE DRAINAGE RESOURCE RECOVERY

Tashane J. Boothe-Lordon[1], Rosemary C. Capo[1], Brian W. Stewart[1], Travis A. Olds[2], Carla E. Rosenfeld[2]

[1] Department of Geology and Environmental Science, University of Pittsburgh, Pittsburgh, PA 15260

[2]Carnegie Museum of Natural History, Pittsburgh, PA 15213

Corresponding Author: Tashane Boothe-Lordon

Email: tjb160@pitt.edu

This PDF file includes:

Supplementary text

Table S6 to S7

Figures S1 to S6

SI References

Supplementary Information Text

Elemental analysis

Samples were analyzed using a computer-controlled (QTEGRA software) Thermo iCAP Q inductively coupled plasma mass spectrometer (ICP-MS) at the Northwestern University Quantitative Bulk-Elemental Information Core (QBIC), Illinois, USA. Blank samples containing 3% nitric acid and dd-H_2O only were also analyzed for quality control. The internal standards used in the analysis were matrix matched to the samples and were added inline using the prepFAST system. The internal standard consisted of 1 ng/mL In and Bi (115In and 209Bi) which was prepared using stock solutions from Inorganic Ventures, Christiansburg, VA, USA. Each sample was acquired using 1 survey run (10 sweeps) and 3 main (peak jumping) runs (40 sweeps). Instrument performance was optimized daily through autotuning followed by verification via a performance report (passing manufacturer specifications). The ICP was also monitored to ensure no carryover/memory effects occurred throughout the run. The detection limits (LOD) and background equivalent concentration (BEC) are shown below in Table S6.

Table S6. Detection limits (LOD) and background equivalent concentration (BEC) Concentration of metals analyzed by ICP-MS.

	55Mn	59Co	60Ni	89Y	139La	140Ce	141Pr	146Nd	153Eu	157Gd	163Dy	172Yb
**LOD**	0.011	0.001	0.032	0.002	0.001	0.001	0.000	0.001	0.000	0.001	0.000	0.000
**BEC**	0.021	0.003	0.083	0.007	0.001	0.004	0.001	0.002	0.000	0.002	0.000	0.000

Data Treatment

Biotic HMO, biomass only, and control experiments. Critical metal adsorption in each experiment was determined by measuring the concentration of metals remaining in solution at each sample point (0.05 h, 3 h, 6 h, 1 d, 4 d, 7 d, 10 d, 18 d, and 31 d). The critical metal concentrations are expected to decrease over time, and metal loss is expected to be primarily due to adsorption by the biotic HMO and/or biomass. Control experiments were used to assess the extent of metal loss in the growth media only, and growth media and aqueous Mn solution only. In the adsorption experiments, we observed a rapid decline in the concentration of all critical metals at the initial sample point, 0.05 h (3 minutes). This resulted in a similar percentage metal loss from solution, particularly for Co and Ni. This pattern of metal loss was also observed in the control experiments (Figure S3-5).

The similarity in the pattern of metal loss suggests that a common adsorption artifact was affecting the metals at the start of the experiments. Artifacts during adsorption experiments can include metal losses during filtration, losses due to precipitation, and losses due to adsorption onto glassware[1] and can result in an overestimation of adsorption. In our experiments, metal loss due to adsorption on filter paper is unlikely since quality control samples did not show reduced metal concentrations after filtration. Despite the low concentration of the metals in these experiments, precipitation may be a potential cause of metal loss. The formation of metal hydroxides has been noted even at low concentrations and underestimated by speciation models.[2] Adsorption to glassware is a common, and most likely adsorption artifact affecting these experiments. Adsorption of trace metals to glassware is known to occur in some adsorption experiments at circumneutral and higher pH values. [1,3,4] We also considered the formation of > 0.22 µm metal-organic complexes with the HEPES buffer as a potential explanation for the high

metal loss observed in the controlled experiments. However, HEPES has been shown to have minimal interaction with metal ions, making it a preferred buffer for maintaining circumneutral pH in cell culture studies.[5] Given its limited metal binding capacity, it is unlikely that the formation of metal-organic complexes with HEPES contributed to the observed metal loss.

To correct for the adsorption data, the amount of metal adsorbed (reported as a percentage) was calculated by subtracting the metal concentration remaining in solution as reported by ICP-MS from the highest concentration of the metal recorded in the "growth media + Mn" control experiment.

Abiotic HMO experiments. The abiotic experiments did not show a pattern of rapid adsorption at the start of the experiment. The amount of REY adsorbed (reported as a percentage) is calculated by subtracting the metal concentration remaining in solution as reported by ICP-MS from the concentrations in the metal spiking solution. Co and Ni concentration in several samples, as reported by ICP-MS, were 1-3.8 μM higher than the spiking solution. The amount of Co and Ni adsorbed (as percentage) was therefore calculated by subtracting the metal concentration remaining in solution from the highest concentration of the metal recorded in the experiments.

Mn concentrations in abiotic HMO experiments. In the abiotic experiments, we observed a rapid increase in dissolved Mn concentration (Figure S6). Some increase in Mn(II) is expected due to redox reactions involving Co(II) and Ce(III) sorption and oxidation [6] and less so Ni sorption.[7] However, in this experiment, the amount of Co(II) and Ce(III) adsorbed does not account for the Mn(II) released. It is possible that the fluctuations in Mn(II) concentrations may be due to complex interactions between the minerals and organic molecules in the growth media over time. Additionally, increases in Mn(II) may have occurred due to the disproportionation of

Mn(III) ions in the structure of H^+ birnessite which can have Mn(III) contents up to 23%, [8] or reduction of Mn(IV) in δ-MnO_2.

Table S7. Concentration of critical metals in spiking solution. The metal concentrations of the spiking solution are typical of Appalachian AMD (sampled at Export, Pennsylvania) with an enrichment factor of 70.

Critical Metal	Concentration (μM)
Y	18.0
La	3.30
Ce	11.4
Pr	1.83
Nd	8.63
Gd	2.7
Dy	2.2
Yb	0.700
**Total REY**	**48.8**
Ni	101
Co	69.0

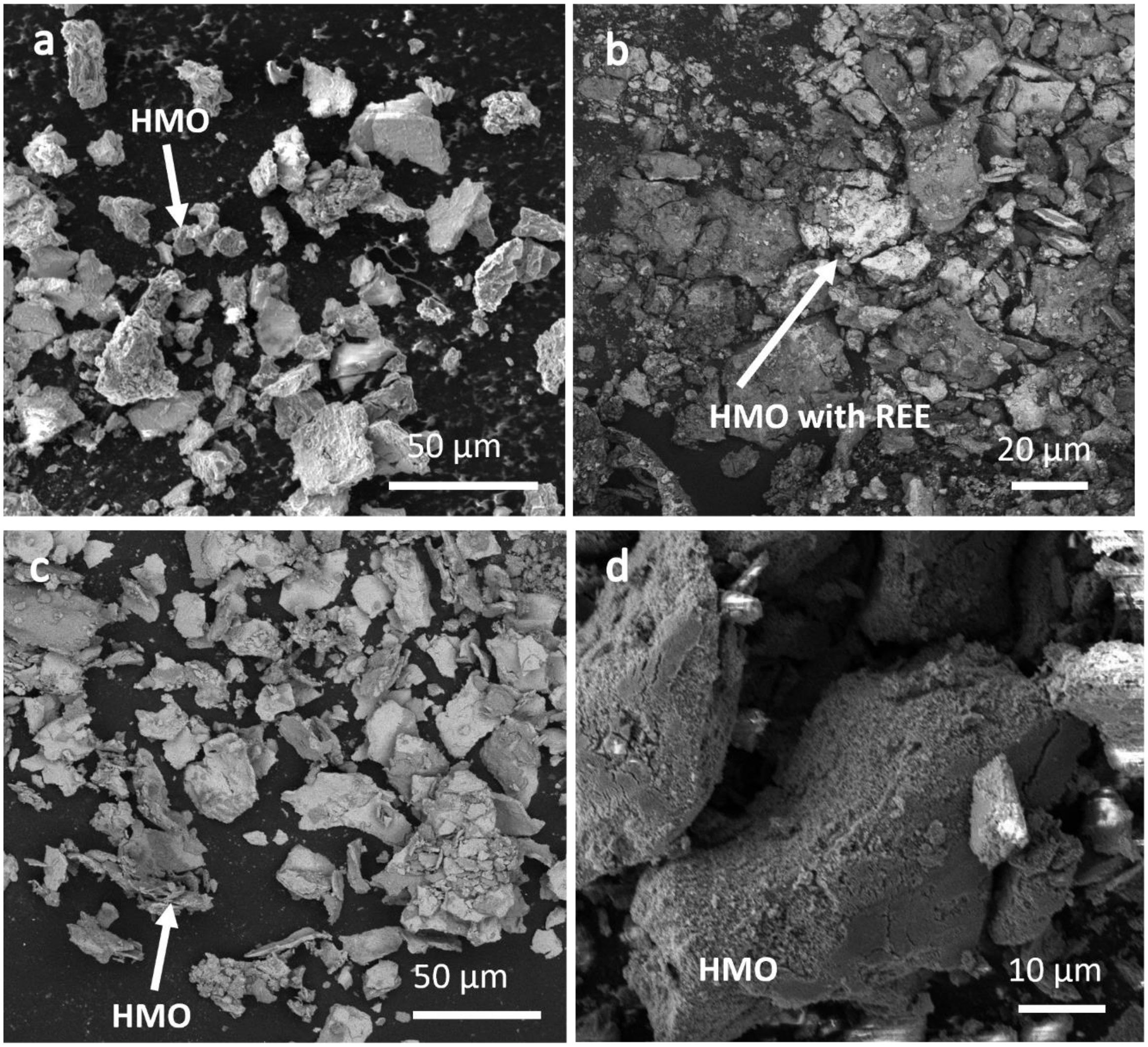

Figure S1. SEM of δ-MnO_2 (**a-b**) and H+ birnessite (**c-d**) 31 days after critical metal addition. Both abiotic HMO minerals are characterized by layered, angular structures with smaller surface area than biotic HMO.

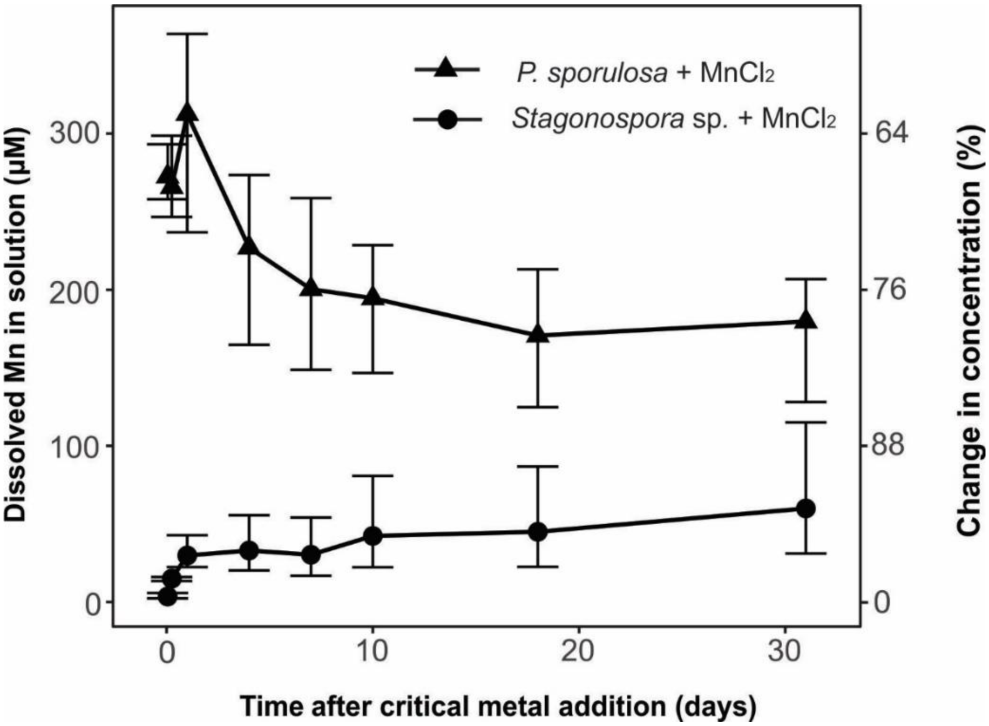

Figure S2. Change in aqueous Mn concentration in biotic experiments after the addition of critical metals. Each point represents the average value for three replicate experiments. The top and bottom of the error bar represent the maximum and minimum values respectively.

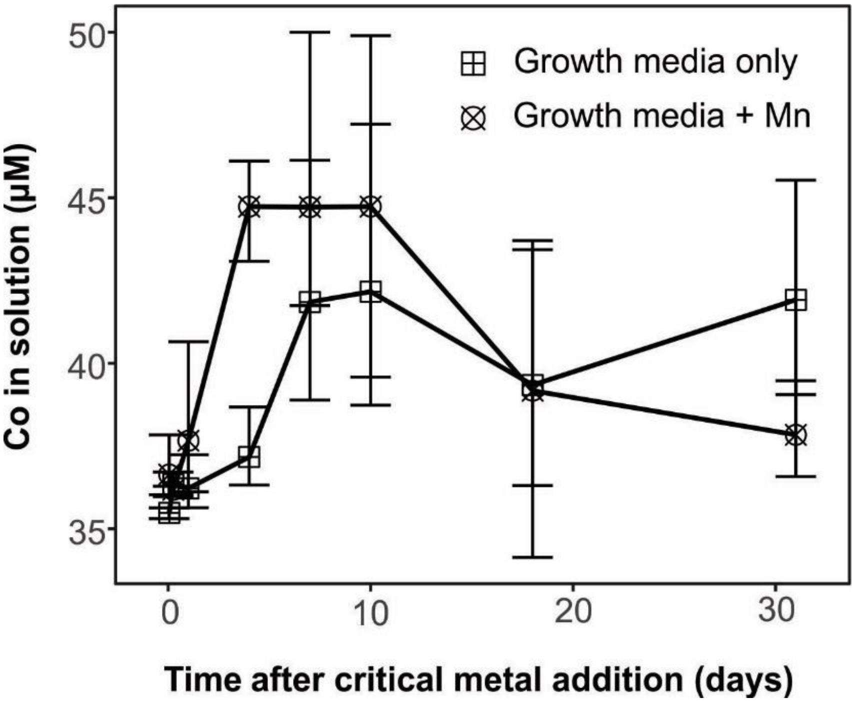

Figure S3. Change in aqueous Co concentration in the control experiments after the addition of critical metals. Each point represents the average value for three replicate experiments. The top and bottom of the error bar represent the maximum and minimum values respectively.

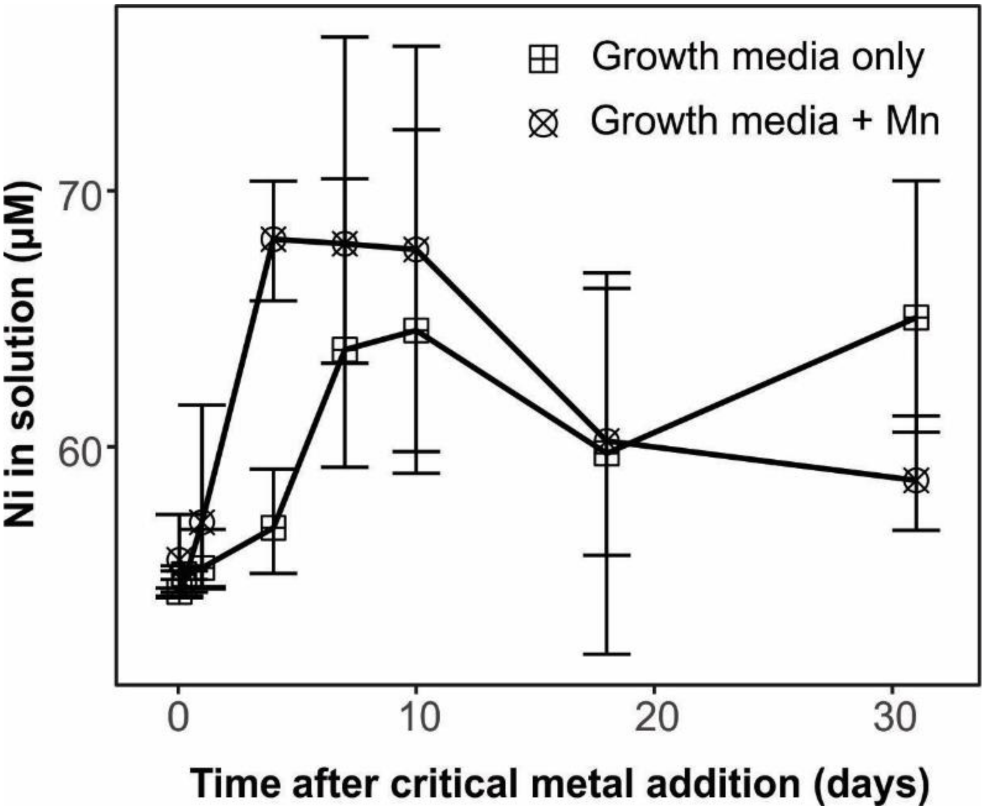

Figure S4. Change in aqueous Ni concentration in the control experiments after the addition of critical metals. Each point represents the average value for three replicate experiments. The top and bottom of the error bar represent the maximum and minimum values respectively.

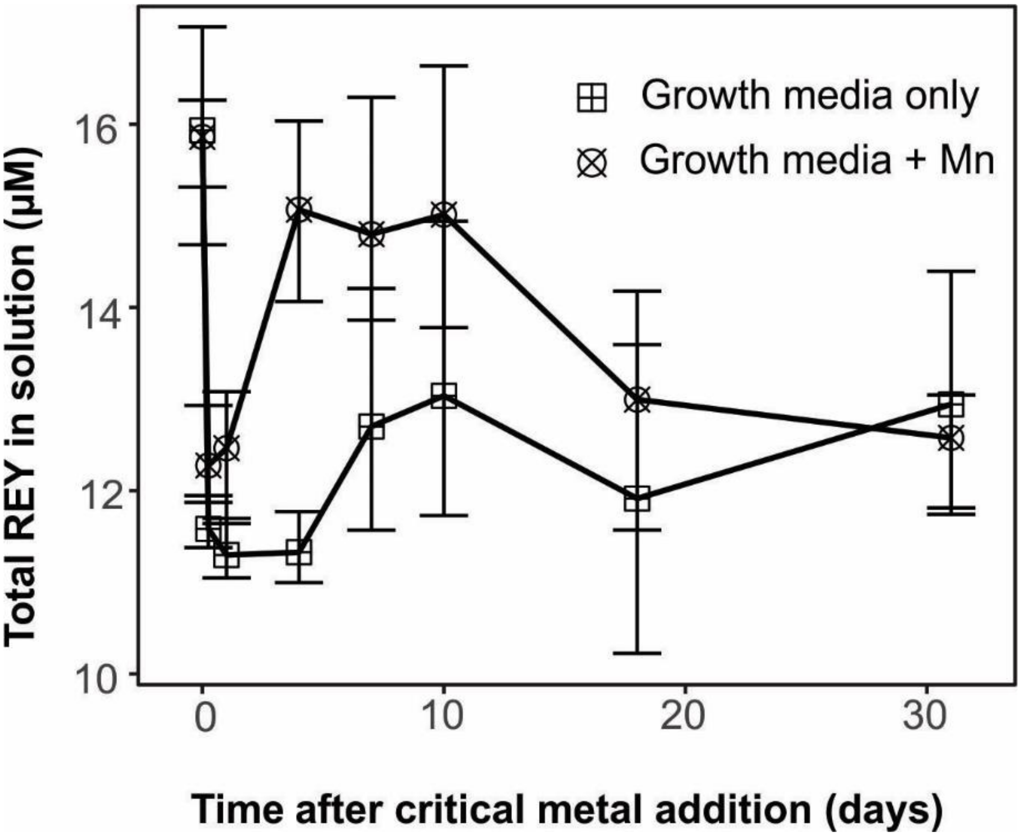

Figure S5. Change in aqueous total REY concentration in the control experiments after the addition of critical metals. Each point represents the average value for three replicate experiments. The top and bottom of the error bar represent the maximum and minimum values respectively.

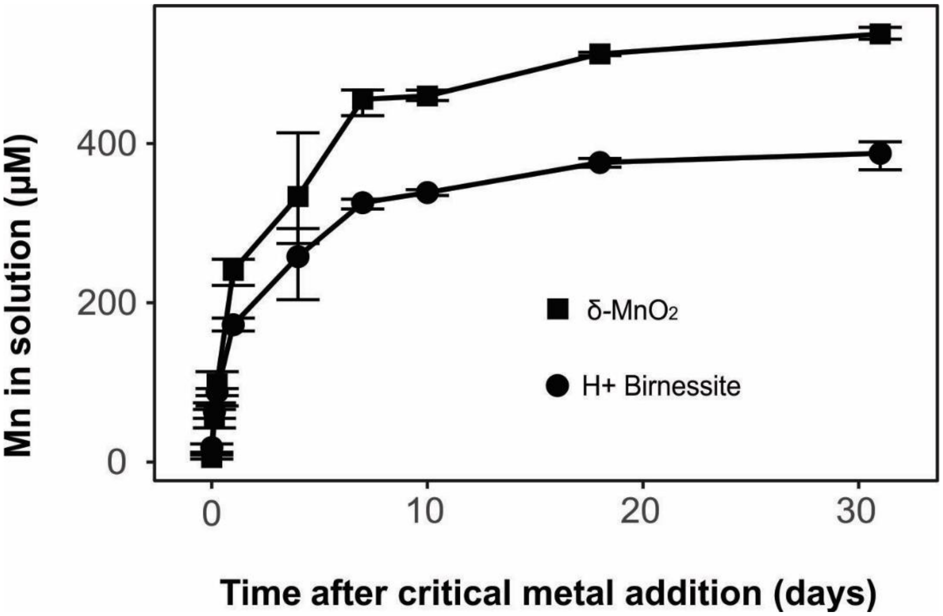

Figure S6. Change in aqueous Mn concentration in abiotic experiments after the addition of critical metals. Each point represents the average value for three replicate experiments. The top and bottom of the error bar represent the maximum and minimum values respectively.

References

(1) Giusti, L.; Taylor, J. H.; Davison, W.; Hewitt, C. Artefacts in sorption experiments with trace metals. *Science of the total environment* **1994**, *152* (3), 227-238.

(2) Haas, S.; Boschi, V.; Grannas, A. Metal sorption studies biased by filtration of insoluble metal oxides and hydroxides. *Science of the total environment* **2019**, *646*, 1433-1439.

(3) Pinheiro, J. P.; Bosker, W. Polystyrene film-coated glassware: a new means of reducing metal losses in trace metal speciation. *Analytical and bioanalytical chemistry* **2004**, *380*, 964-968.

(4) Fischer, A.; Kroon, J.; Verburg, T.; Teunissen, T.; Wolterbeek, H. T. On the relevance of iron adsorption to container materials in small-volume experiments on iron marine chemistry: 55Fe-aided assessment of capacity, affinity and kinetics. *Marine Chemistry* **2007**, *107* (4), 533-546.

(5) Good, N. E.; Winget, G. D.; Winter, W.; Connolly, T. N.; Izawa, S.; Singh, R. M. Hydrogen ion buffers for biological research. *biochemistry* **1966**, *5* (2), 467-477.

(6) Zhao, H.; Feng, X.; Lee, S.; Reinhart, B.; Elzinga, E. J. Sorption and oxidation of Co (II) at the surface of birnessite: Impacts of aqueous Mn (II). *Chemical Geology* **2023**, 121281.

(7) Simanova, A. A.; Kwon, K. D.; Bone, S. E.; Bargar, J. R.; Refson, K.; Sposito, G.; Peña, J. Probing the sorption reactivity of the edge surfaces in birnessite nanoparticles using nickel (II). *Geochimica et Cosmochimica Acta* **2015**, *164*, 191-204.

(8) Villalobos, M.; Toner, B.; Bargar, J.; Sposito, G. Characterization of the manganese oxide produced by Pseudomonas putida strain MnB1. *Geochimica et Cosmochimica Acta* **2003**, *67* (14), 2649-2662.
